# The Source of Scarlet Fever Poison

**Published:** 1871-09

**Authors:** 


### The Source of Scarlet Fever Poison.

Whilst we know much of its mode of operation upon the body, we remain profoundly ignorant of the nature and source of the poison of scarlatina, and he must be a bold man who will venture to erect, upon our present stock of data, a theory as to its origin. Dr. Carpenter, of Croydon, however, who speaks with great authority on sanitary matters, has broken fresh ground on this point, and, in a very interesting and suggestive paper read to the Medical Society, brought forward a series of facts which, he believes, go far to show that the poison of scarlet fever is an emanation resulting from incipient decomposition of the blood of vertebrated animals, either healthy or diseased, and produced under the influence of certain conditions of temperature, magnetic state, and moisture; this poison being more virulent, perhaps, when produced from diseased blood. Dr. Carpenter detailed the history of various outbreaks of scarlet fever occurring in localities where slaughter-house refuse had been freely used as manure or in houses contiguous to places at which it had been stored, or such as were so placed as to receive emanations from decomposing blood. Dr. Carpenter did not deny the contagiousness of scarlet fever, but he argued for its origination as it were *de novo,* in the manner indicated. He further stated that, though the enforcement of ordinary sanitary precautions, such as are now in force, did not prevent the occurrence of the disease, yet it very materially influenced the mortality; and he adduced a number of facts, which he gathered from the condition of Croydon, to show that in those houses from which sewer emanations were rigorously excluded, and in which ventilation and cleanliness were observed, scarlatina was robbed of its virulence, and became a mild affection; where as in the badly-sewered and badly-built portions of the town the disease was very fatal. He expressed the belief that scarlatina would be shorn of its fatality if blood were not allowed to be shed in our town and villages except under proper regulations by which it should be excluded from the sewers, and our houses were probably hygiened. Such is the gist of Dr. Carpenter's paper.—*London Lancet.*

